# Exercise and the Cardiovascular System

**DOI:** 10.1155/2012/210852

**Published:** 2012-05-31

**Authors:** Saeid Golbidi, Ismail Laher

**Affiliations:** Department of Pharmacology and Therapeutics, Faculty of Medicine, University of British Columbia, Vancouver, BC, Canada V6T 1Z3

## Abstract

There are alarming increases in the incidence of obesity, insulin resistance, type II diabetes, and cardiovascular disease. The risk of these diseases is significantly reduced by appropriate lifestyle modifications such as increased physical activity. However, the exact mechanisms by which exercise influences the development and progression of cardiovascular disease are unclear. In this paper we review some important exercise-induced changes in cardiac, vascular, and blood tissues and discuss recent clinical trials related to the benefits of exercise. We also discuss the roles of boosting antioxidant levels, consequences of epicardial fat reduction, increases in expression of heat shock proteins and endoplasmic reticulum stress proteins, mitochondrial adaptation, and the role of sarcolemmal and mitochondrial potassium channels in the contributing to the cardioprotection offered by exercise. In terms of vascular benefits, the main effects discussed are changes in exercise-induced vascular remodeling and endothelial function. Exercise-induced fibrinolytic and rheological changes also underlie the hematological benefits of exercise.

## 1. Introduction

The American College of Cardiology/American Heart Association recommends at least 30 minutes of moderate (at 50–70% of maximal predicted heart rate) exercise on most days to reduce the risk of cardiovascular events [1]. Several human studies clearly demonstrate that chronic aerobic exercise regimens improve cardiovascular function. This is true not only in healthy subjects without any underlying risk factors [2], but also in older people [3], and those with cardiovascular risk factors [4]. Indeed, those with cardiovascular risk factor/disease will benefit more. There is a much higher consistency in the results of studies which assess participants with cardiovascular disease/risk factors compared to healthy subjects. Patients with hypertension [5], type 2 diabetes [6], metabolic syndrome [7], stable cardiovascular disease [8], myocardial infarction [9], and congestive heart failure [10], all benefit from exercise training compared to those who do not participate in any training. Importantly, an exercise regimen that improves endothelial function in diabetic patients fails to benefit healthy subjects [6, 11]. In healthy individuals, a longer and more intense exercise protocol is needed to induce measureable changes in cardiovascular parameters, while older and sicker subjects can benefit from less intense exercise regimens.

Treatment and control of established known cardiovascular risk factors includes the reduction of hypercholesterolemia, hypertension, and smoking [12]. During the past decade, the mortality rates from coronary heart disease and stroke in the United States were reduced by more than 25%. However, the prevalence of diabetes mellitus has increased steadily, mostly because of an epidemic of adiposity [13]. This unfortunate change can mitigate further improvements in cardiovascular mortality and can potentially reverse the decline in cardiovascular disease incidence that has been achieved through decades of education, improved health care, and better lifestyle choices.

Prevention can be categorized into three components. Primary prevention is concerned with health promotion activities, which prevent the actual occurrence of a specific illness or disease. Secondary prevention promotes early detection or screening and treatment of disease and limitation of disability. This level of prevention is also called health maintenance. Tertiary prevention is directed at recovery or rehabilitation of a disease or conditions after the disease has developed. Physical activity, as one the most important components of cardiovascular disease prevention, has crucial roles at all three levels. Despite the strong evidence linking physical activity to cardiovascular disease risk reduction, there remains much uncertainty regarding the underlying mechanisms. In this paper, we discuss the benefits of exercise as a modifiable lifestyle parameter and its relation to cardiovascular health at molecular level. We will discuss recent findings related to the cardiovascular benefits of exercise and also survey the clinical evidence for exercise-induced cardiovascular improvement.

## 2. Cardiac Effects of Exercise

### 2.1. Boosting Antioxidant Levels

Free radicals, which are a subset of reactive oxygen species (ROS), are physiological byproducts of aerobic metabolism [14] and are widely recognized for their dual roles as both deleterious and beneficial species, since they can be either harmful or beneficial to living systems [15]. High concentrations of free radicals harm living organisms through reactions with adjacent molecules such as proteins, lipids, carbohydrates, and nucleic acids. As a result, mammalian cells have evolved a variety of antioxidant mechanisms to control ROS production and propagation [16]. On the other hand, mild oxidative stress can act as a stimulant of physiological antioxidant systems and as a trigger for various physiological adaptations [17]. This has led to our current understanding of free radical-mediated effects of exercise as a phenomenon of hormesis [18], according to which there may be a bell-shaped curve of oxidative stress in response to exercise, with none and excessive exercise being considered harmful and moderate levels being of most beneficial [19, 20]. Regular physical exercise delays the accumulation of ROS-mediated cell damage by improving the antioxidative protective mechanisms in the myocardium. The strongest evidence to directly link increases in myocardial antioxidants and exercise-induced cardioprotection implicates a contributory role for manganese superoxide dismutase (MnSOD). It is generally believed that even short-term endurance exercise results in a rapid increase in myocardial MnSOD activity [21–23], as shown in studies using antisense oligonucleotide techniques to silence MnSOD genes and so prevent exercise-induced increases in myocardial MnSOD activity [22, 24]. Yamashita et al. [22] reported that inhibition of exercise-induced increases in cardiac MnSOD abolished protection against myocardial infarction, findings that were confirmed by Hamilton et al. [25] who concluded that MnSOD plays a key role against ischemia-reperfusion-(I/R-) induced cardiac arrhythmias.

## 3. Role of Exercise in Reducing Inflammation by Decreasing in Epicardial Fat

Ectopic fat refers to the accumulation of triglycerides within cells of non-adipose tissue; these tissues normally contain only small amounts of fat. Visceral areas, liver, heart and/or muscle are common sites for deposition of ectopic fat [26]. The amount of epicardial fat is directly related to the increases in visceral fat [27, 28], insulin resistance [27, 29], triglyceride levels and blood pressure [27, 29], and in general with the metabolic syndrome [29]. Accumulation of epicardial fat is also important in the pathogenesis of cardiovascular diseases. There are multiple reasons to support the concept that epicardial and perivascular adipose tissue are important in inducing atherosclerosis [30, 31]. Firstly, there is close anatomical proximity between epicardial fat and coronary vessels. There is no fibrous fascial layer to impede diffusion of free fatty acids and adipokines between adipose tissue and the underlying coronary arteries and myocardium [28]. This can lead to lipotoxicity and development of cardiomyopathy [32]. Increased intra-cardiomyocyte triglycerides in diabetic patients is associated with impaired left ventricular diastolic function independent of age, body mass index, heart rat, visceral fat, and diastolic blood pressure [33].

The role of adipose tissue in secreting metabolically active substances is well established. It is believed that a balance between anti-atherosclerotic adipokines such as leptin and adiponectin and pro-atherosclerotic cytokines, such as IL-6, TNF-*α*  and monocyte chemotactic protein-1 (MCP-1) adjusts metabolic and cardiovascular homeostasis at local and remote sites. Mazurek et al. showed inflammatory properties of cardiac fat by a paired sampling of epicardial and subcutaneous adipose tissues before the initiation of cardiopulmonary surgery [34]. Higher levels of IL-1*β*, IL-6, MCP-1 and TNF-*α*  mRNA and protein were observed in epicardial adipose stores irrespective of clinical variables such as diabetes, BMI, and drug use. On the other hand, visceral fat obesity is associated with decreased concentrations of insulin-sensitizing and anti-inflammatory adipokines [26].

A study by Kim et al. evaluated the effects of aerobic exercise (without diet restriction) on ventricular epicardial fat thickness. They showed that ventricular epicardial fat thickness was reduced significantly after aerobic exercise training and was also associated with decreases in visceral adipose tissue. Exercise caused a greater loss of epicardial fat than it to reduce BMI, and body weight [35]. Exercise also reduces waist circumference and causes losses in abdominal and visceral fat, even in the absence of any loss of body weight, in both men and women regardless of age [36]. Therefore, increased physical activity lowers secretion of pro-inflammatory adipokines that is related to reducing the amount of fat stored in abdominal depots.

## 4. Heat Shock Proteins (HSPs)

The heat shock response is a common cellular reaction to external (stressful) stimuli such as ischemia [37], hypoxia [38], acidosis [39], oxidative stress [40], protein degradation [41], increased intracellular calcium [42], and energy depletion [43]. It is generally accepted that exercise increases the expression of cardiac HSPs. The mechanistic link between exercise and myocardial expression of HSPs is unclear. However, a variety of stresses associated with exercise, including heat stress and hypoxia, reduced intracellular pH, reactive oxygen and nitrogen species production, depletion of glucose and glycogen stores, increase in cytosolic calcium levels and cardiomyocyte stretching can all contribute to HSP elevation in cardiac muscle [44]. Increased expression of HSP70 in cardiomyocytes is associated with increased cell survival and protection against ischemic damage [45]. The HSP70 response is reduced with ageing, which is consistent with a diminished endurance to stress in the elderly [46].

## 5. Endoplasmic Reticulum Stress Proteins

These are a family of cardioprotective proteins collectively termed endoplasmic reticulum (ER) stress proteins which help cellular homeostasis by maintaining intracellular calcium regulation and protein folding during an I/R injury [47]. The two most important ER stress proteins are Grp78 and Grp94 (which belong to the HSP family) and are overexpressed in cultured cardiomyocytes during oxidative stress and calcium overload [48]. Since overexpression of these ER stress proteins provides ER protection during an I/R insult, it may be that these proteins contribute to exercise induced cardioprotection. However, studies by Murlasits et al. demonstrate that at least short-term exercise training does not elevate ER stress proteins, and therefore, short-term exercise-induced cardioprotection may not be linked to ER stress adaptation [49].

## 6. Mitochondrial Adaptation

There is an important role for mitochondria in myocardial I/R injury. Exercise training results in cardiac mitochondrial adaptations that result in decreased ROS production, increasing their ability to tolerate high calcium levels. Reductions in ROS production could be related to decreased superoxide production or increased mitochondrial antioxidant enzyme activity. A study by Judge et al. [50] indicated that MnSOD activity was significantly lowered in subsarcolemmal and interfibrillar mitochondria, leading to the suggestion this may reflect a reduction in mitochondrial superoxide production. However, this issue is currently a matter of considerable debate.

Mitochondria of exercised animals are able to tolerate higher levels of calcium. Mitochondria isolated from hearts of exercised animals are more resistant to calcium-induced mitochondrial permeability transition pore (mPTP) opening [51]. Furthermore, exercise training induces a mitochondrial phenotype that is protective against apoptotic stimuli [52]. These changes include increases in the protein levels of primary antioxidant enzymes in both subsarcolemmal and interfibrillar mitochondria, attenuation of ROS-induced cytochrome c release, reduced maximal rates of mPTP opening (*V*_max⁡_), prolonged time to *V*_max⁡_ in both subsarcolemmal and interfibrillar mitochondria, and increased levels of anti-apoptotic proteins including the apoptosis repressor with a caspase recruitment domain. These results are consistent with the concept that exercise induced mitochondrial adaptations contribute to exercise induced cardioprotection and are in keeping with our study on the effect of exercise on renal mitochondria in diabetic mice [53].

Exercise also induces a down regulation of mitochondrial monoamine oxidase-A (MAO-A). Bianchi et al. showed that H_2_O_2_ production by MAO-A plays a critical role in post I/R events that lead to cardiac damage [54]. Thus MAO-A knockout mice demonstrate higher level of protection against I/R-induced cardiac damage, which was also related to significantly lower levels of ROS generation [55]. Exercise also significantly reduces MAO-A protein levels in both cardiac subsarcolemmal and inter-myofibrillar mitochondria [56].

## 7. Role of Sarcolemmal Potassium Channels

The sarcolemmal K_ATP_ channels are a potential target for exercise induced I/R protection. During ischemia, heart cells become energy depleted, which leads to increased anaerobic glycolysis to compensate for ATP depletion. The resulting acidosis increases the influx of Na via the Na/H exchanger and inhibits the ATP-dependent sarcolemmal Na/K ATPase to augment the initial accumulation of Na [57]. The high intracellular Na concentration prompts the Na/Ca exchanger to work in the reverse mode, producing cytosolic and mitochondrial Ca overload [58]. Upon reperfusion, a burst of ROS is generated by mitochondria, while intracellular Na overload continues as a result of the impaired function of Na/K ATPase. It was Noma [59] who initially hypothesized that opening of sarcolemmal K_ATP_ channels induced by hypoxia, ischemia, or pharmacological openers of the K_ATP_ channel shortens the cardiac action potential duration by accelerating phase III repolarization. An enhanced phase 3 repolarization would inhibit Ca entry via L-type channels and prevent cellular Ca overload. Furthermore, the slowing of depolarization would also reduce Ca entry and slow or prevent the reversal of the Na/Ca exchanger. These actions would increase cell viability via a reduction in Ca overload during ischemia and early reperfusion. There is considerable experimental support for the protective role of sarcolemmal K_ATP_ channels in myocardial function [60–64].

## 8. Role of Mitochondrial Potassium Channels

Several studies confirm the role of mitochondrial K channels in protection against I/R injury [65–67]. Prostacyclin analogs protect cardiac myocytes from oxidative stress mainly via activation of type 3 prostaglandin E_2_ receptors during I/R injury. Activation of these receptors primes the opening of mitochondrial K_ATP_ channels [68]. However, there is some controversy regarding the role of mitochondrial K_ATP_ channels in exercise preconditioning of the heart. For example, Domenech et al. reported that the early effect of exercise preconditioning of the heart is mediated through mitochondrial K_ATP_ channels [69], while Brown et al. reported that mitochondrial K_ATP_ channels are not required for exercise-induced protection against I/R-induced myocardial infarction [70]. It has also been recently suggested that mitochondrial K_ATP_ channels provide antiarrhythmic effects as part of exercise-induced cardioprotection against I/R injury [71]. It should be mentioned that the molecular characteristics of mitochondrial K_ATP_ channels remains elusive and that additional research is needed to clarify their function in cardiac function.

## 9. Cyclooxygenase II and Exercise Induced Cardioprotection

The phenomenon of ischemic preconditioning whereby brief episodes of sublethal ischemia renders the myocardium resistant to subsequent ischemic stressoccurs in two phases: (i) an early phase that starts within a few minutes after the initial ischemic stimulus, lasts for 2-3 h, and is due to adenosine and bradykinin release and (ii) a second phase, which begins 12–24 h later and lasts for 3-4 days [72, 73]. This later phase of ischemic preconditioning is caused by the simultaneous activation of multiple stress responsive signaling pathways, including COX-2 and the inducible form of nitric oxide synthase (iNOS), resulting in the heart developing a phenotype that confers sustained protection against both reversible and irreversible myocardial I/R injury [73]. Similar to ischemic stimuli, both short- (1–3 days) and long-term (weeks to months) exercise protocols are equally effective in conferring cardioprotection against I/R injury [21, 74].

## 10. Vascular Effects of Exercise

The etiology of nearly all of the lifestyle-related vascular diseases can be narrowed down to endothelial dysfunction. The vascular endothelium consists of a monolayer of cells that line all the internal surfaces of cardiovascular system and plays a critical role in regulation of vascular homeostasis [75]. The endothelium plays a vital role regulating arterial dilation and constriction by manufacturing vasodilator [nitric oxide (NO), prostacyclin (PGI2), endothelium-derived hyperpolarizing factor (EDHF)] and vasoconstrictor [endothelin-1 (ET-1), platelet-activation factor (PAF)] agents [76]. A key component of intact endothelial function is NO production by endothelial nitrous oxide synthase (eNOS), which incorporates oxygen into L-arginine. The anti-inflammatory, vasodilatory and platelet inhibitory effect of NO have important roles in the maintenance of vascular hemostasis [77]. Hence, endothelial function measurements are considered useful surrogate end points in clinical research [78], especially since decreased endothelium-derived NO bioavailability has an independent prognostic value for adverse cardiovascular events in the presence of risk factors but without clinically apparent coronary artery disease [79–81] or established coronary atherosclerosis [82–85]. In some studies, the risk of cardiovascular events such as myocardial infarction or ischemic stroke was 3-4 folds higher in cardiovascular patients with endothelial dysfunction compared to those with a normal endothelial function [85–87].

## 11. Exercise and Endothelial Function

Physical activity increases vascular expression of eNOS both in animals and human beings [88–91]. The importance of this phenomenon has been confirmed in patients with stable coronary artery disease and chronic heart failure [92, 93]. There are several reports suggesting that exercise-induced up-regulation of vascular eNOS expression is closely related to the changes of frequency and the intensity of physical forces within the vasculature, especially shear stress. Exercise-induced increases in heart rate will augment cardiac output and vascular shear stress, leading to increased expression of eNOS [88]. Increased NO synthesis secondary to amplified shear stress induces extracellular superoxide dismutase (SOD) expression in a positive feedback manner so as to inhibit the degradation of NO by ROS [94].

Another parallel mechanism that participates to this harmony is upregulation of eNOS through exercise induced ROS production, since exercise-induced increases in shear stress stimulates vascular production of ROS by an endothelium dependent pathway [95]. Endothelial NADPH oxidase has a critical role in this process [96]. Superoxides are rapidly converted to H_2_O_2_ by SOD; hydrogen peroxide then diffuses through the vascular wall and increases the expression and activity of eNOS [97, 98]. Thus, increased expression of SOD1 and SOD3 (which facilitate the generation of hydrogen peroxide from superoxide), augments the effect of hydrogen peroxide on exercise induced eNOS expression. On the other hand, eNOS expression is not increased in catalase overexpressing transgenic mice [89, 99].

Another putative mechanism is exercise-induced increases in arterial compliance which is mediated by reduction of plasma ET-1 concentration as well as the elimination of ET-1 mediated vascular tone. Twelve weeks of aerobic exercise training results in increased arterial compliance, which was accompanied by decreased plasma ET-1 levels. Moreover, the increase in central arterial compliance observed with ET-receptor blockade before the exercise intervention was eliminated after the exercise training intervention [100]. These results indicate that endogenous ET-1 participates in the mechanisms underlying the beneficial influence of regular aerobic exercise on central arterial compliance.

## 12. Exercise Induced Vascular Remodeling

Exercise training has a significant impact on the morphology of various blood vessels. These structural changes are followed by functional changes and lead to improved blood flow. Exercise induces “angiogenesis”, which is an expansion of the capillary network by the formation of new blood vessels at the level of capillaries and resistance arterioles, and arteriogenesis, which is an enlargement of existing vessels [101].

### 12.1. Angiogenesis 

It has been speculated that endurance exercise stimulates angiogenesis by either a division of preexisting endothelial cells or by bone marrow-derived endothelial progenitor cells and monocyte or macrophage derived angiogenic cells [102]. Some reports indicate that physical activity improves the mobilization of endothelial progenitor cells in healthy subjects and in patients with cardiovascular risk and coronary artery disease [103, 104]. Indeed, angiogenesis is regulated by a net balance between positive (angiogenic) and negative (angiostatic) regulators of blood vessel growth. A balance favoring predominantly positive regulators are an angiogenic phenotype whereas a shift favoring negative regulators is an angiostatic phenotype. Therefore, an impaired regulation of angiogenesis is often associated with the development of angiogenesis-dependent diseases such as atherosclerosis.

Endostatin is an endogenous angiostatic factor identified originally in conditioned media of murine hemangioendothelioma cells [105, 106]. Several studies show that the proteolytic release of endostatin from collagen XVIII is mediated by proteases of many classes, such as cysteine proteases, matrix metalloproteases, and aspartic proteases [107, 108]. The potent antiangiogenic effects of endostatin are mediated via a combination of effects on endothelial cells where endostatin inhibits cellular proliferation and migration and stimulates apoptosis [109, 110]. The biological effects of endostatin are mainly attributed to its antagonism of vascular endothelial growth factor (VEGF) signaling [111]. Angiogenesis has both beneficial and deleterious effects in atherosclerosis. While increased angiogenesis in cardiac tissue may be a favorable sign in the healing of the ischemic tissues [112], progressive angiogenesis in a primary atherosclerotic lesion could be a cause of plaque expansion [113, 114]. There are several studies showing that exercise induces a local angiogenic phenotype characterized by overexpression of VEGF in skeletal muscle [115] and heart [112]. This phenomenon can prevent ischemia in these tissues. Exercise can also exert beneficial effects against atherosclerosis by increasing circulating endostatin, which inhibits development of atherosclerotic plaque by blocking angiogenesis in the plaque tissue [116]. Endurance activity improves angiogenesis by reducing endostatin plasma levels [117]. Even though the different exercise protocols in these experiments can explain these discrepant results, further studies are needed to elucidate the precise mechanisms.

### 12.2. Arteriogenesis

Exercise training increases the diameter of large arterioles, small arteries, and conduit arteries. Another important aspect of exercise-induced changes in capillarity is the onset and persistence of exercise-induced arteriogenesis. The induction of arteriogenesis is an important vascular adaptation [118], since arteriogenesis leads to the formation of large conductance arteries capable of compensating for the loss of function of occluded arteries. Animal studies and clinical observations provide evidence for a significant correlation between regular physical exercise and increased coronary artery lumen diameter [119, 120]. In one study, an 8-week training program increased the contractile response to low doses of dobutamine in patients with chronic coronary artery disease and having a left ventricular ejection fraction below 40%. This implies that short-term exercise training can improve quality of life by improving left ventricular systolic function during mild to moderate physical activity in patients with ischemic cardiomyopathy [121]. Moreover, eight patients with coronary heart disease and exertional angina pectoris successfully completed a 11–15 week program of endurance exercise conditioning. Angina threshold was determined by upright bicycle ergometer exercise and by atrial pacing. The product of heart rate and arterial systolic blood pressure at the exercise angina threshold was higher after conditioning, suggesting that conditioning increased the maximum myocardial oxygen supply during exercise [122].

## 13. Anti-Inflammatory Effect of Exercise in Vascular Tissue

Inflammation has a prominent role in the pathogenesis of several cardiovascular diseases. Atherosclerosis is an inflammatory disease that is mediated by monocyte derived macrophages which accumulate in arterial plaques and become activated to release cytokines that cause tissue damage [101]. As evidence accumulates favoring the role of inflammation during the different phases of atherosclerosis, it is likely that markers of inflammation such as high sensitivity C-reactive protein (hs-CRP) may be increasingly used to provide additional insights on the biological status of atherosclerotic lesions. CRP is considered to be an independent predictor of cardiovascular events and of the outcome of acute coronary syndromes [123]. Besides its role as a marker of systemic inflammation and a predictor of cardiovascular risk, CRP and other inflammatory cytokines also directly trigger vascular dysfunction [124], possibly via altering calcium channel expression and activity [125], upregulation of Rho-kinase expression and function [126], increasing the production of ROS [127], and/or enhancing cyclooxygenase expression [128]. In turn, cyclooxygenase enzymes cause vascular hypercontractility by increasing the synthesis of constrictor prostanoid(s) [129, 130] and excessive formation of ROS [131].

Exercise produces a short-term inflammatory response that is accompanied by leukocytosis, increases in oxidative stress, and plasma levels of CRP. This pro-inflammatory response is followed by a long term anti-inflammatory effect [132]. Regular exercise reduces CRP, IL-6, and TNF-*α*  levels and also increases anti-inflammatory substances such as IL-4 and IL-10 [133, 134]. In healthy young adults, a 12-week high-intensity aerobic training program down regulates cytokine release from monocytes [134]. In fact, even leisure time physical activity (e.g., walking, jogging, or running, etc.) reduces hs-CRP concentration in a graded manner [135]. Subjects with higher baseline CRP levels (>3.0 mg/L) will benefit more [136–138].

## 14. Hematological Benefits of Exercise

Exercise in humans is associated with a number of hematological changes. For instance, Bonsignore et al. [139] reported a higher number of circulating hematopoietic progenitor cells in runners, indicating modulation of bone marrow activity by habitual running. Regular exercise training also augments the number of endothelial progenitor cells in patients with cardiovascular risk factors and coronary artery disease and is associated with improved vascular function and NO synthesis [140]. Exercise-induced increased mobilization of hematopoietic stem and progenitor cells, endothelial progenitor and circulating angiogenic cells may have a role in physiologic repair and/or compensatory mechanisms toward promotion of angiogenesis and vascular regeneration [102, 141]. Exercise also increases hemoglobin, hematocrit, platelet numbers, and interleukin-6 levels in young healthy individuals of both genders and all fitness levels which propose a role for exercise in enhancing tissue repair mechanisms [142].

Another hematological effect of exercise is on the rheological properties of the blood. Hemorheology is the study of flow properties of blood and its elements [143]. Abnormal hemorheology is considered an independent risk factor for cardiovascular disease and has an important role in the etiology of atherothrombogenesis. There are a limited number of studies showing increases in blood viscosity following a variety of exercise protocols. This effect has been attributed to increases in hematocrit and plasma viscosity [144]. However, cross-sectional and longitudinal studies indicate that trained athletes have more dilute blood which is secondary to expanded blood volume, particularly plasma volume [145–147]. This discrepancy may partially be explained by shear stress. At low shear stress rates, increased hematocrit leads to increased effective cell volume and blood viscosity, while at high shear rates, increased hematocrit enhances red cell deformation which in turn reduces effective cell volume and therefore compensates for increased viscosity [148]. It has been shown that fit patients have a lower blood and plasma viscosity, fibrinogen concentration, and red blood cell aggregation [148]. Enhanced blood fluidity can facilitate oxygen delivery to the exercising muscles due to decreased resistance to blood flow within the microcirculation.

**Figure 1:**
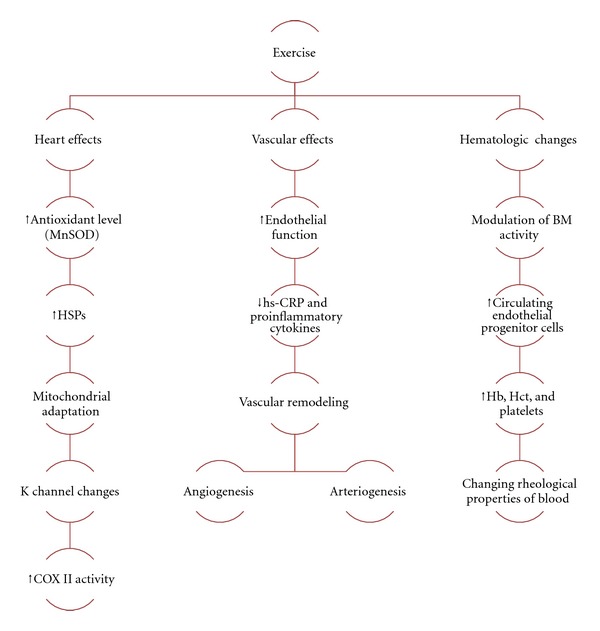
Selected effects of excise on heart, vessels and blood components of cardiovascular system.

De Paz et al. measured different components of fibrinolytic system in runners and control groups before and after exercise. Acute maximal exercise resulted in elevation of fibrinolysis, as shown by higher levels of fibrin degradation products and fibrinogen degradation products, in both groups. The increased fibrinolytic activity was higher in trained individuals, which could have resulted from higher tissue plasminogen activator release and reduced formation of “tissue plasminogen activator-plasminogen activator inhibitor complexes” [149]. In spite of this report, it seems that the results of different studies on exercise and hemostatic function have been biased by several confounding variables, such as age, exercise protocol, or time and methods for hemostatic evaluation. Most studies show a transient hypercoagulable state after acute and exhaustive physical activity. This could explain increased thrombotic events and sudden death during or immediately after exercise. These changes, however, appear to be reversible after a few hours, offering some protection, particularly in trained individuals, against the risk of thrombosis and adverse cardiovascular events [150]. This antithrombotic effect of chronic exercise is also reversible and will return to previous values within 4 weeks of exercise cessation [151, 152].  Figure 1 summarizes the mentioned effects of exercise on cardiovascular system.

## 15. Clinical Evidence

Increased levels of physical activity and fitness, both in men and women, reduce the relative risk of death by about 20–35% [153, 154]. Some studies even suggest greater benefits (up to 50% risk reduction) for exercise in terms of all-cause mortality and death from cardiovascular disease [155]. Blair et al. in an eight-year followup study evaluated physical fitness and risk of all-cause and cause-specific mortality in a large number of healthy men and women. The lowest quintiles of physical fitness were associated with significant higher risk of death from any cause compared with the top quintiles [156]. Lee and Skerrett reviewed 44 observational studies to determine the dose-response relation between physical activity and all-cause mortality. They reported an inverse dose-response relation between volume of physical activity and all-cause mortality rate; thus a 1000 Kcal/week was associated with significant 20–30% risk reduction [157]; these studies were later confirmed by others [158, 159]. Most experts currently encourage a minimum amount of exercise that uses one 1000 Kcal per week and acknowledge increased benefits of higher energy expenditures. It should be reiterated, however, that lower levels of energy expenditure are also associated with health benefits [159–161]. A systematic review by Oguma and Shinoda-Tagowa showed that there is a graded inverse relationship between physical activity and cardiovascular adverse events where a minimum of one hour walking per week (and possibly less) has protective effects [162].  Table 1 summarizes recent clinical studies on the cardiovascular benefits of exercise.

## 16. Summary

There is great interest in changes as a means to effectively reduce cardiovascular disease risks. In particular, physical activity has been widely studied because of its well-known effects on the metabolic syndrome, insulin sensitivity, cardiovascular disease risks, and all-cause mortality. The detailed molecular mechanisms for these favorable effects remain unknown and continue to be actively investigated at various levels. Of the many findings reported, it is clear that modifications of oxidative stress have an important role in the cardiovascular protection offered by exercise.

Among the proposed mechanisms for exercise-induced cardiac effects, changes in mitochondrial function and sarcolemma K_ATP_ channel regulation play significant roles. Indeed, mitochondria are important determinants of survival in cardiac myocytes exposed to I/R. Thus modifications in mitochondrial function by exercise can greatly impact on cardiac muscle. In the vasculature NO is a major role player: the anti-inflammatory, vasodilatory, and platelet inhibitory effects of NO are indispensable for the maintenance of vascular hemostasis. Exercise increases the expression and activity of eNOS, likely by changes in shear stress, and so modulates the production of NO. Besides the vascular and metabolic effects of NO, it also possesses a number of physiological properties, which makes it a cardioprotective molecule in the setting of myocardial I/R injury. Exercise-induced increases in arterial compliance, which is mediated by reduction of plasma ET-1 concentration and has an impact on vascular morphology, are among other speculated mechanisms for vascular changes in trained subjects. Exercise also exerts anti-inflammatory effect in both cardiac and vascular compartments. An increased number and mobilization of hematopoietic stem cells, endothelial progenitor, and angiogenic cells as well as rheological alterations are hematological component of this harmonized concert.

Further studies are clearly warranted so that we can gleam a better understanding of the mechanisms of exercise as a preventive and therapeutic measure for the cardiovascular system. An additional benefit is that by so doing, we will better customize appropriate levels of physical training for individual patients.

## Figures and Tables

**Table 1:** Selected clinical trials on the cardiovascular effects of exercise.

Reference	Patient groups and characteristics	Intervention and followup	Measured parameters	Outcome
[163]	50 hypertensive patients divided in 2 groups and stratified for other variables (a) Routine antihypertensive therapy (b) Antihypertensive therapy plus 6 month exercise.	(i) Incremental CPET on a bicycle ergometer 30 min a day for 6 months.	Peak_*V*_O_2___, power_max_, AT, *V*_O_2_AT_, *t*_AT_, HR_rest_, LAVI, E/A ratio, DT, IVST, Ea/Aa ration.	Peak_*V*_O_2___, power_max_, AT, *V*_O_2_AT_, *t*_AT_ were increased, HR_rest_ decreased and LAVI, E/A ratio, DT, IVST, Ea/Aa ration improved in exercise group.

[164]	98 patients with moderate to severe (*n* = 34), mild (*n* = 33) and preserved (*n* = 31) LVEF that were randomized to: (a) Exercise training plus usual care. (b) Usual care alone.	Exercise training on a treadmill or bicycle ergometer three times a week for 6 months.	LVEF, E/A ratio, DT.	Exercise tolerance and LVEF increased in exercise group, ↑ E/A ratio and ↓ DT in patients with mild and preserved LVEF. ↓ E/A ratio and ↑ DT in patients with moderate to severe systolic dysfunction and advanced diastolic dysfunction.

[165]	496 old people categorized base on their daily physical activities (a) <4 hr weekly (b) 4 hr weekly (c) At least 1 hr daily (d) Sport at least twice weekly.		Echocardiographic assessment of cardiac structure and function.	Mean EF was lower among sedentary versus active women. No other significant differences (systolic or diastolic function) were observed.

[166]	64 patients with HFpEF randomized to: (a) Endurance/resistance training plus usual care (b) Usual care alone.	Supervised, facility-based training program consisting of endurance and resistance training (32 sessions).	(i) Changes in *V*_O_2__ after 3 months. (ii) cardiac structure, diastolic function and Qol.	*V*_O_2__ increased, E/e and left atrial volume index decreased in ET group. Physical functioning score improved with ET group.

[167]	365 sedentary, overweight, hypertensive, postmenopausal women randomly assigned to: (a) Sedentary controls (b) Exercise groups at: (a) 4 Kcal/kg/week (b) 8 Kcal/kg/week (c) 12 Kcal/kg/week.	Exercise group patients underwent 50% (4 Kcal/kg/week), 100% (8 Kcal/kg/week), or 150% (12 Kcal/kg/week) of the NIH-CDP physical activity guideline.	Time and frequency domain indices of HRV.	Parasympathetic indices of HRV increased in women that were >60 years old.

[168]	34 patients with stable symptoms of intermittent claudication randomized to: (a) Strength training (ST) (b) Walking training (WT).	ST consisted of eight exercises, 3 sets of 10 repetitions, intensity of 11–13 on 15 grade Borg scale. WT consisted of walking on treadmill, 15 bouts of 2 min, intensity of 11–13 on grade Brog scale.	Resting systolic BP, HR, rate-pressure product, maximal exercise time.	Resting systolic BP, HR and rate pressure product decreased in both groups. Submaximal systolic BP and rate-pressure product also decreased in both groups. Maximal exercise time increased in both groups.

[169]	29 patients with stable chronic MI were assigned to: (a) Training group (*n* = 17) (b) Control (*n* = 12).	Exercise intensity set at 55–70% of *V*_O_2_max⁡_, subjects perceived exertion rating of 12-13 Borg scale, 3 bouts a week for 12 weeks	Myocardial perfusion study.	Exercise induced perfusion changes in the infarct zone is proportional to the amount of residual viable myocardium.

[170]	26 young healthy subjects assigned to: (a) Training group (*n* = 13) (b) Control group (*n* = 13).	The subjects performed LSR twice a week at 50% of one repetition maximum for 10 weeks. Training consisted of 5 sets of ten repetitions with an interest rest period of 30 s.	Changes in baPWV and FMD.	FMD increased and baPWV decreased in exercise group.

[171]	38 type II diabetic patients were assigned to: (a) Exercise group (*n* = 21) (b) Control group (*n* = 17).	Exercise group received 3–5 bouts a week for 3 months, each bout consisted of 75 min combination of aerobic and resistance exercise.	Endothelial function (by FMD), insulin resistance, adipocytokines and inflammatory markers.	BMI decreased while *V*_O_2__ and FMD were significantly increased in exercise group (changes in Hb_A1C_, LDL and HDL cholesterol, adiponectin, hsCRP were similar in both groups).

[172]	37 patients with CHF randomly assigned to: (a) Exercise training group (b) Sedentary.	12 weeks of exercise (20–30 min a day) on a bicycle ergometer adjusted to the work load of 50–60% of *V*_O_2_max⁡_.	*V*_O_2_max⁡_, LVEF, number and functional capacity of CPC, FMD, and capillary density in skeletal muscles.	Exercise training improved *V*_O_2_max⁡_, LVEF, FMD, CPC number and function also increased capillary density in skeletal muscles.

[173]	44 health young FH+ women, assigned to: (a) AIT (*n* = 16) (b) CMT (*n* = 16) (c) Controls with FH+ (*n* = 12) 15 healthy young women with normotensive parents and negative FH as the 2nd control group.	Exercise protocol consisted of 60 min (AIT or CMT) endurance exercise 3 times a week for 16 weeks.	ABP, insulin, insulin sensitivity, carotid-femoral PWV, NE, ET-1, NO_*x*_.	AIT and CMT were equally effective in improving ABP, insulin and insulin sensitivity. AIT was superior in improving cardiovascular fitness, BP, NE, ET-1 and NO_*x*_ response.

[174]	44 pre-pubertal obese children were randomly assigned to: (a) Exercise group (*n* = 22) (b) Control group (*n* = 22) (c) 22 lean matched controls.	The exercise group trained 60 min 3x a week for 3 months, then both groups trained twice per week for another 3 months.	BP, IMT, FMD, BMI, body fat, *V*_O_2_max⁡_, physical activity and biological markers were assessed at 3 and 6 months.	After 3 months: significant differences in BP, BMI, abdominal fat, and *V*_O_2_max⁡_. After 6 months: significant changes changes in arterial stiffness and IMT were significant.

ABP: ambulatorial blood pressure; AIT: high-intensity aerobic interval training; AT: anaerobic threshold; baPWV: brachial ankle pulse wave velocity; BMI: body mass index; CMT: moderate-intensity continuous exercise training; CPC: circulating progenitor cells; CPET: cardiopulmonary exercise test; DT: deceleration time of the mitral E wave; E/A ratio: peak mitral filling velocities during early (E) and late (A) diastole; E/e ratio: the ratio of mitral velocity to early diastolic velocity of the mitral annulus; Ea/Aa ratio: tissue Doppler indices mean; EF: ejection fraction; Et: exercise training; ET-1: endothelin-1; FH+: positive family history of hypertension; FMD: brachial flow mediated dilation; HDL: high density lipoprotein; HFpEF: heart failure with preserved ejection fraction; HR_rest_: heart rate at rest; HRV: heart rate variability; hsCRP: high sensitivity C-reactive protein; IMT: arterial intima-media thickness; IVST: interventricular septum thickness in diastole; LAVI: left atrial volume index; LDL: low density lipoprotein; LSR: low intensity resistance training with short inter-set rest period; LVEF: left ventricular ejection fraction; NE: norepinephrine; NIG-CDP: national institutes of health consensus development panel;  NO_*x*_: nitrite/nitrate level; PWV: pulse wave velocity; Qol: qullity of life; *t*_AT_ time from beginning to anaerobic threshold; *V*_O_2__ volume of consumed oxygen; *V*_O_2_AT_: volume of consumed oxygen at anaerobic threshold; *V*_O_2_max⁡_: maximal oxygen consumption.
